# Dried Whole Black Soldier Fly Larvae Consumption Supports Gestation, Lactation, and Growth in Cats

**DOI:** 10.3390/ani15081078

**Published:** 2025-04-08

**Authors:** Ian J. Banks, Daniel Adams, Jabarry R. Belgrave, Elizabeth A. Lewis, Elizabeth A. Koutsos

**Affiliations:** 1 EnviroFlight, LLC, Apex, NC 27359, USA; ian.banks@enviroflight.net (I.J.B.); dr.daniel.adams.ii@gmail.com (D.A.); 2 NutraSteward, Ltd., Frederick House, Johnston, Haverfordwest SA62 3AQ, UK; jabarry.belgrave@nutrasteward.com (J.R.B.); elizabeth.lewis@nutrasteward.com (E.A.L.)

**Keywords:** dried whole black soldier fly larvae, pet food, regulatory authorization, cats, gestation, lactation, kittens, growth

## Abstract

This study evaluated the consumption of dried whole black soldier fly larvae (DBSFL) to meet the nutritional requirements of gestating and lactating cats and their kittens post-weaning. Queens (*n* = 8/treatment) were randomly assigned to a control diet or diets containing 20% DBSFL through gestation and lactation. Weaned kittens (*n* = 8/treatment) were maintained on their dams’ diet for 10-weeks. There were no differences (*p* > 0.05) in the litter size, dietary intake, body weight, or fecal quality of the queens during the trial. The kittens receiving the DBSFL diet consumed more on an absolute or caloric basis than those fed the control diet at week 5 (*p* = 0.1) and week 10 (*p* = 0.03) and had higher body weights at weeks 6 through 10 (*p* < 0.05). The blood and urinalysis parameters were generally within normal ranges, with some variations observed between treatments that were not considered biologically significant. The dry matter, crude protein, and amino acid digestibility levels were higher in kittens fed the DBSFL diet (*p* = 0.05). These data are the first to demonstrate the safety and efficacy of DBSFL for the gestation and lactation of queens and for the growth of kittens, as well as its suitability in diets for cats of all life stages.

## Simple Summary

Insect-derived ingredients are sustainable sources of premium protein and energy for the pet food industry, although their safety and efficacy are yet to be established in the most sensitive life stages of cats and dogs. The goal of this study was to evaluate the consumption of dried whole black soldier fly larvae, as a source of protein and energy components, in these sensitive life stages of cats, including in pregnant and lactating cats, and their offspring. In the study, adult female cats—before, during, and after pregnancy—and their weaned kittens received either a control diet or a treatment diet that partially replaced chicken meal and poultry fat with dried whole black soldier fly larvae. Throughout the duration of the study, cat and kitten health and development were maintained. The dried whole black soldier fly larvae diet was also found to be more digestible by kittens than the control diet. This study is the first to demonstrate the safety and efficacy of dried whole black soldier fly larvae, and its protein and energy components, when fed to pregnant and lactating cats and their kittens.

## 1. Introduction

Insect-derived ingredients are of increasing interest to the pet food industry, providing an opportunity for the bioconversion of feedstocks of low or no value into premium protein and energy ingredients, while having a favorable environmental footprint [1,2].

For any ingredient, an evaluation of its safety and efficacy is critical prior to market entry and is required to gain regulatory authorization in different jurisdictions. The safety of insect ingredients for use as nutrient sources in animal feed, including black soldier fly larvae (BSFL)-based ingredients, has been extensively reviewed in the published literature [3,4,5,6,7,8,9]. The successful demonstration of the safety of larvae (BSFL) ingredients by certain manufacturers in the US has resulted in the Association of American Feed Control Officials (AAFCO) ingredient definitions for BSFL ingredients, in the form of dried whole black soldier fly larvae (DBSFL), partially defatted BSFL meal (DBSFL meal), and BSFL oil. These authorizations are for several species, including finfish, poultry, swine, adult dogs, and adult cats (pending) for DBSFL and DBSFL meal; and finfish, poultry (pending), swine, adult dogs, and adult cats for BSFL oil. The current US authorization [10] explicitly requires that the BSFL be raised on feed-grade materials, which minimizes the risk of chemical and microbiological hazards accumulating in BSFL ingredients. This authorization does limit the use of some potential feedstocks (e.g., post-consumer food waste) but allows for the use of pre-consumer by-products that may otherwise have low or no value.

It is important to recognize that BSFL-based ingredients can vary in nutrient composition and quality (e.g., digestibility) due to the BSFL diet, life stage at harvest, and processing methods used to create these ingredients. Unlike more traditional ingredients, there is significant potential for variation in quality and composition due to the insect rearing program. The nutrient composition of the BSFL will be significantly influenced by the feedstock [11,12]; notably, the fatty acid, mineral, and heavy metal profiles can vary dramatically due to different feeding programs. Similarly, the life stage of the BSFL impacts the nutrient composition and digestibility of those nutrients [13]. For example, in a previous study, the fat content was low early post-hatch, then increased, then declined again as the BSFL entered the pre-pupal and pupal life stages, although the digestibility of that fat was highest from the pre-pupal and pupal life stages. The crude protein and amino acid contents also varied substantially between life stages, indicating that the age of processing can have a significant impact on the overall nutritional value of BSFL ingredients. The processing methods may also impact the nutritional quality of BSFL, as illustrated by a study that found that DBSFL meal produced from oven-dried BSFL had significantly higher in vitro digestibility compared to meals produced from BSFL that were blanched and then dried or those that were microwaved [14].

The potential use of BSFL ingredients in pet food is supported by numerous in vitro and in vivo evaluations in relevant animal models. In adult dogs, several trials have demonstrated the safety and efficacy of BSFL ingredients as protein and energy sources for adult dogs at up to 30% inclusion of DBSFL meal and up to 5% inclusion of BSFL oil. In these trials, the dogs maintained their body weight (BW), feed intake, and fecal scores, and the BSFL ingredients generally had similar or slightly lower nutrient digestibility compared to traditional animal protein sources [15,16,17,18,19,20]. Overall, these data support the safety and efficacy of BSFL-derived ingredients as alternatives to traditional protein and energy sources in adult dog diets. Where differences have been observed, such as in digestibility parameters, these may be related to the diet used to rear the BSFL or the processing method used to produce the finished BSFL-derived ingredients.

There are also several published feeding trials in cats. Adult cats fed for 70 days on diets containing either 5% DBSFL partially replacing poultry by-product meal and poultry fat, 10% DBSFL meal replacing poultry by-product meal, or 1.5% BSFL oil replacing poultry fat had similar intake, fecal output, hematology, and blood clinical chemistry results across all treatments, and preferred the BSFL-based diets over the control diet [21]. As seen in dogs, the protein digestibility was slightly lower than in poultry-based diets. Similar results were seen in cats fed up to 35% DBSFL meal for 6 weeks [22] and in cats fed 4% DBSFL meal in semi-purified diets. Together, these data support the safety and efficacy of BSFL-derived ingredients in adult cats at maintenance.

While the utility in maintenance diets for dogs and cats is well-documented, the ability of BSFL ingredients to sustain gestation, lactation, and growth has not yet been demonstrated, although numerous trials have demonstrated the acceptability of BSFL ingredients for piglet growth [23,24,25]. The nutritional requirements of these life stages are more demanding than those of adult animals and the diets for these animals require a separate evaluation [10]. The purpose of this research was to evaluate the safety and efficacy of the protein and lipid components of DBSFL created using a specific feeding program and processing method, to support the gestation and lactation of female cats, as well as the growth of their kittens post-weaning.

## 2. Materials and Methods

### 2.1. BSFL Description

The DBSFL used in this trial was derived from 4th–5th instar larvae raised on a proprietary blend of bakery materials and distillery by-products from the bourbon industry, which were also oven-dried (“EnviroBug”, EnviroFlight LLC, Maysville, KY, USA). The nutrient composition of the DBSFL reared on this feedstock and with this method of drying is provided in Table 1.

### 2.2. Cat Trials

A gestation, lactation, and growth study was conducted at Ontario Nutri Labs, Inc. (Fergus, ON, Canada), a registered research animal facility. Prior to the start of the study, a palatability test was performed to ensure that all potential queens accepted both the control and the DBSFL diets. A total of 25 queens were fed both control and DBSFL diets for 5 days. All queens consumed both the control and DBSFL diet, and from that cohort 16 queens were selected for the full trial and randomly assigned to the control or DBSFL diet (*n* = 8/treatments; Table 2).

The diets were formulated to be isocaloric and isonitrogenous and to meet or exceed AAFCO and National Research Council (NRC) recommendations for gestation, lactation, and growth [26,27]. The DBSFL partially replaced chicken meal and poultry fat, as well as some brewers rice and corn, in the control diet. To minimize bias, the identity of the control and DBSFL diet subjects were blinded to the staff responsible for conducting the study.

All queens were initially fed the control diet for a 7 day acclimatization period and then offered their assigned dietary treatment prior to breeding. The queens (approximately 3.4 kg; at least 1 year old, in at least their 2nd heat) were fed diets containing either 0% (control) or 20% DBSFL during the entire gestation (approximately 10 weeks, weeks 0 to 9) and lactation (approximately 6 weeks, weeks 10 to 15) phases. The cats were fed based on their resting energy requirements based on calculations of their body weight and the energy density of their diet prior to the study starting, whereas after breeding, the queens were fed ad libitum, as energy requirements can vary based on litter size and individual variation [28]. Fresh water was provided ad libitum. Following parturition, the kittens remained with their dams until weaning (approximately 6 weeks of age).

The cats were group housed for the majority of the study but were housed individually for feeding and study-related purposes where individual data collection, such as urine and stool collection, was required. All rooms were environmentally controlled for lighting, consisting of natural lighting via windows and LED full-spectrum lighting consistent with a natural light cycle, as well as for temperature and ventilation, and were sized in accordance with the Animal Welfare Acts of the US and Canada. A full veterinary physical exam of the queens (including the body condition score (BCS) and haircoat scoring) was undertaken during the acclimation period (baseline), at mid-gestation, within 24 h post-parturition, and at the end of the lactation period. The BWs of the queens were recorded weekly and their food consumption was recorded daily. Blood samples were taken from the queens at baseline (during acclimation period), mid-gestation, post-parturition (5 to 7 days), and at weaning. Prior to blood collection, the queens were fasted for 16 h, which was reduced to 8 h during gestation to limit the time the food was withheld. Their blood samples were analyzed for hematology (red blood cell (RBC), hematocrit, hemoglobin, mean corpuscular volume (MCV), mean corpuscular hemoglobin (MCH), mean corpuscular hemoglobin concentration (MCHC), red cell distribution width (RDW), % reticulocyte, reticulocyte, reticulocyte hemoglobin, white blood cell (WBC), % neutrophil, % lymphocyte, % monocyte, % eosinophil, % basophil, neutrophil, lymphocyte, monocyte, eosinophil, basophil, and platelet) and serum biochemistry (albumin, total protein, globulin, albumin/globulin ratio, total bilirubin, conjugated bilirubin, aspartate aminotransferase (AST), alanine transaminase (ALT), alkaline phosphatase (ALP), gamma-glutamyl transferase (GGT), creatine kinase, creatinine, blood urea nitrogen (BUN), glucose, total carbon dioxide (TCO_2_; bicarbonate), magnesium, sodium, potassium, sodium/potassium [Na/K] ratio, calcium, phosphorus, symmetric dimethylarginine test (SDMA), chloride, cholesterol, triglyceride, amylase, lipase, hemolysis index, icterus index, and lipemia index) data (IDEXX Laboratories Canada, Markham, ON, Canada). Their whole blood and plasma taurine were measured, as well using standard amino acid extraction protocols and a Biochrom 30 amino acid analyzer (UC Davis Amino Acid Lab, Davis, CA, USA).

Stool and urine samples were collected as free catch samples using a 3-tier collection litter box system concurrently with blood samples. The urine was analyzed for its color, protein, glucose, creatinine, ketone, bilirubin, pH, and specific gravity (by refractometer) data, and the urine protein/creatinine ratio (UPCR) was determined, as well as a microscopic sediment examination to identify crystals, casts, red and white blood cells, and epithelial cells (IDEXX Laboratories Canada, Markham, ON, Canada). The stool quality was assessed using the WALTHAM scale, in which each stool was rated on a scale of 1 to 5, with 1 being “bullet-like” and 5 being moist and loose, and with 3 being an optimal score [29]. Since the urine and stool collection involved being away from group housing for prolonged periods, all cats on collection protocols received supervised enrichment with staff for at least 2 h during these periods.

Upon parturition, the kittens received a full veterinary physical examination (including BCS and haircoat scoring) within 24 h of birth, and again at weaning. Each kitten’s BW was recorded weekly during the lactation phase.

To examine the most rapid period of development and growth of the kittens fed the DBSFL-based diet, a subsample of kittens (*n* = 8 per dietary treatment, representing at least 3 unique litters and with equal gender distribution) were maintained on the diet assigned to their dam for an additional 10 weeks post-weaning. All kittens were less than 9 weeks old at the time of inclusion in the growth trial and were fed the same diet assigned to their dam between the end of the lactation phase and the start of the growth phase. Their body weights were measured at the start of the growth phase and monitored at seven-day intervals until the end of the 10-week trial. Their blood and urine were collected as described for queens at the start of the growth trial (18–20 days post-weaning), and after 5 and 10 weeks on the growth trial. The blood hematology, clinical chemistry, and urinalysis were assessed as described for the queens. The kittens received a full veterinary physical exam (including BCS and haircoat scoring) at the start of the growth trial (post-weaning), at the mid-point of the growth trial (5 weeks), and at the end of the trial (10 weeks).

### 2.3. Nutrient Digestibility

Nutrient digestibility was measured via an analysis of diet nutrient concentrations after grinding (dry matter (AOAC 930.15), ash (AOAC: 942.05), crude protein (AOAC: 2001.11), crude fiber (AOCS: Ba6a-05), crude fat (AOAC: 954.02), and calories (Parr Caloric Bomb); UC Davis Amino Acid Lab, Davis, CA, USA). Upon study initiation, a representative sample of each diet was taken, ground, and homogenized independently by the ONL Analytical Lab, and then analyzed for dry matter (AOAC: 930.15), ash (AOAC: 942.05), crude protein (AOAC: 2001.11), crude fiber (AOCS: Ba6a-05), crude fat (AOAC: 954.02), and calorie (Parr Caloric Bomb) data. Samples of both diets were analyzed for amino acids (AOAC 994.12 and AOAC 988.15; UC Davis Amino Acid Lab, Davis, CA, USA). The results from the analyses were used to calculate the digestibility of the diets.

All feces voided throughout days 67 to 71 (120 h) of the growth study were collected. Fecal samples for each cat were weighed and frozen daily for subsequent analyses. The collected feces were dried at 47 °C, weighed, ground, and analyzed for dry matter, ash, crude protein, crude fiber, crude fat, calorie, and amino acid data, as described for the diet analysis. The nitrogen-free extract (NFE%) or carbohydrate data were calculated using the following formula:

(1) $$NFE\%=100-\left(\%\;crude\;protein+\%\;crude\;fat+\%\;crude\;fiber+\%\;moisture+\%\;ash\right)$$

and the estimated metabolizable energy (ME) was calculated using the following formula:

(2) $$ME\left(kcal/kg\right)=10\times \left[\left(3.5\times crude\;protein\right)+\left(8.5\times crude\;fat\right)\;+\right.\left.\left(3.5\times nitrogen\;free\;extract\right)\right]$$

For better accuracy, the quantitative collection method without urine collection was used to calculate the metabolizable energy using the fecal and feed analysis data and the following formula:

(3) $$ME\;\left(kcal/kg\right)=\left(\frac{\left(a\times b\right)-\left(c\times d\right)-\left[\left(b\times e/100\right)\right.-\left.\left(d\times f/100\right)\right]\times g}{b}\right)\times 1000$$

where a = gross energy of food (kcal/g), b = weight of food consumed (g), c = gross energy of feces in 100% of dry matter (kcal/g), d = weight of feces collected in 100% of dry matter (g), e = percentage of protein consumed as feed (%), f = percentage of protein in the feces in 100% of dry matter (%), and g = correction factor for energy lost in urine (cat) = 0.86 kcal/g.

### 2.4. Statistical Analysis

The jamovi 2.6.13 statistical software (www.jamovi.org; accessed on 6 January 2025) with the GALMJ: General Analyses for the Linear Model in jamovi 3.4.2 module was used to analyze all data. The individual queens and kittens were utilized as the experimental units. The litter size and number of still births were analyzed via Kruskal–Wallis tests due to failing the normality assumptions of analysis of variance (ANOVA) tests. The macronutrient and amino acid digestibility were analyzed via an ANOVA, with diet as a factor. All other data were analyzed via linear mixed models with time (timepoint or week) and diet as factors, and the animal ID as a random effect. The residuals from the statistical models were explored using histograms, quantile–quantile plots, and Shapiro–Wilk and Kolmogorov–Smirnov normality tests. Natural log and square root transformations were used for normality violations, although few variables violated normality of residuals assumptions and transformations did not improve the distribution fit of these variables. Therefore, the data were analyzed as is. Planned comparisons were subsequently conducted to compare the treatment diets at every time. Mean, standard error of the mean (SEM), and probability (*p*) values were reported for each analysis. Statistical significance was defined as *p* ≤ 0.05 and statistical trends were defined as *p* > 0.05 and ≤0.10.

## 3. Results

The veterinary examinations performed during the gestation–lactation phase of the trial showed the queens to be in good physical health. Upon trial commencement, fifteen queens were given the BCS of ideal (5), while one queen, randomly assigned to the DBSFL diet, had a BCS of overweight (6). One queen was removed from the control group as a result of having two kittens that were underdeveloped and had likely died during early pregnancy. Another queen was removed from the DBSFL diet group during the lactation phase of the study as a result of having a large, overdue kitten resulting in a cesarian section and subsequent rejection of the kitten. The kitten was successfully fostered by another queen receiving the 20% DBSFL diet and remained in the trial through the lactation phase but was not included in the growth trial. At the end of the gestation–lactation phase of the study, thirteen queens were given a BCS of ideal (5) and one DBSFL-fed queen had a BCS of underweight (4). All surviving kittens were assessed to be in good physical health within 24 h of birth, and at weaning all kittens were given a BCS of ideal (5). The average litter size from the control queens was 3.63 ± 0.46 and from the DBSFL-fed queens was 2.50 ± 0.46, with no significant difference between treatments (*p* = 0.11). Similarly, the numbers of stillborn kittens did not differ between treatments (0.38, 0.25 ± 0.257 for control and DBSFL-fed queens, respectively, *p* = 0.71).

The diet intake, body weight, and fecal quality of the queens during the gestation and lactation phases are presented in Table 3. The dietary intake of the queens fed the DBSFL diet tended to be lower at weaning on an absolute intake basis (*p* = 0.06) but was not significantly different from the control-fed queens at any other timepoint. The energy intake values (on a kcal per day basis) of the queens fed the DBSFL diet were lower than those fed the control diet but only reached statistical significance at weaning (*p* = 0.02). The body weights were similar between treatments over time. The fecal quality levels were also consistent between treatments but the queens fed the DBSFL diets tended to have slightly higher and more optimal fecal scores (i.e., closer to a score of “3”) post-partum (*p* = 0.07).

The blood hematology parameters at all timepoints were generally within normal ranges (see Supplementary Materials Table S1), except for the reticulocyte hemoglobin (14.8, 14.9, and 14.4 pg at mid-gestation, post-partum, and weaning, respectively, vs. 15.3–22.9 pg) and WBC (20.9 × 10^9^/L at weaning vs. 3.9–19.0 × 10^9^/L) counts in the cats receiving the control diet, which varied slightly from the reference ranges (Table 4). For blood hematology parameters with no significant impact or trend for an impact of diet, data are not presented.

There were a few differences in blood hematology due to dietary treatment. The mean corpuscular volume tended to be higher in the cats fed the DBSFL diets at baseline (*p* = 0.09) and mid-gestation (*p* = 0.08) and was significantly higher at weaning (*p* = 0.05) relative to the controls. The mean corpuscular hemoglobin volume tended to be higher in the queens fed the DBSFL diets at weaning (*p* = 0.07). Compared to the controls, the absolute number of reticulocytes was higher in the queens fed the DBSFL diet at mid-gestation (*p* = 0.05), and the reticulocyte hemoglobin volume tended to be higher in the queens fed the DBSFL diet at the post-partum sampling (*p* = 0.09) and weaning (*p* = 0.05) points. The white blood cell numbers were higher in the queens fed the control diet at weaning (*p* < 0.01). The platelet counts tended to be higher in the control-fed queens at mid-gestation (*p* = 0.07) and were significantly higher at weaning (*p* = 0.04).

The blood clinical chemistry parameters of the queens were generally within normal ranges (see Supplementary Materials Table S2) for all parameters throughout the trial, except for glucose, which was lower than the reference range at the post-partum sampling point (3.5 and 3.7 mmol/L vs. 4.0–9.7 mmol/L), and SDMA, which was higher than the reference range at baseline (14.1 and 14.9 μg/dL vs. 0–14 μg/dL) and in the post-partum period (17.0 and 17.8 μg/dL vs. 0–14 μg/dL), irrespective of the treatment (control or DBSFL-containing diets). For blood clinical chemistry parameters with no significant impact or trend for an impact of diet, data are not presented.

The dietary treatment affected the cats’ globulin levels, with the queens fed DBSFL diets tending to have higher globulin levels at mid-gestation (*p* = 0.09) and significantly higher globulin levels at post-partum sampling (*p* = 0.03) relative to the controls. The alkaline phosphatase levels also tended to be higher for the DBSFL-fed queens at weaning (*p* = 0.06).

The whole blood taurine levels in the queens were maintained within the reference ranges [30] and did not differ significantly between treatments throughout the gestation and lactation periods (mean whole blood taurine 416.89 ± 12.600 nmol/mL; mean plasma taurine 126.26 ± 6.454 nmol/mL). There were no differences in urine pH, urine specific gravity, or urinary creatinine due to dietary treatment (see Supplementary Materials Table S3). The urinary protein level was significantly (*p* = 0.04) higher at post-partum sampling for the queens fed the DBSFL diet compared to those fed the control diet (65.00 and 40.45 mg/dL, respectively) but the results did not differ at any other timepoint.

**Figure 1:**
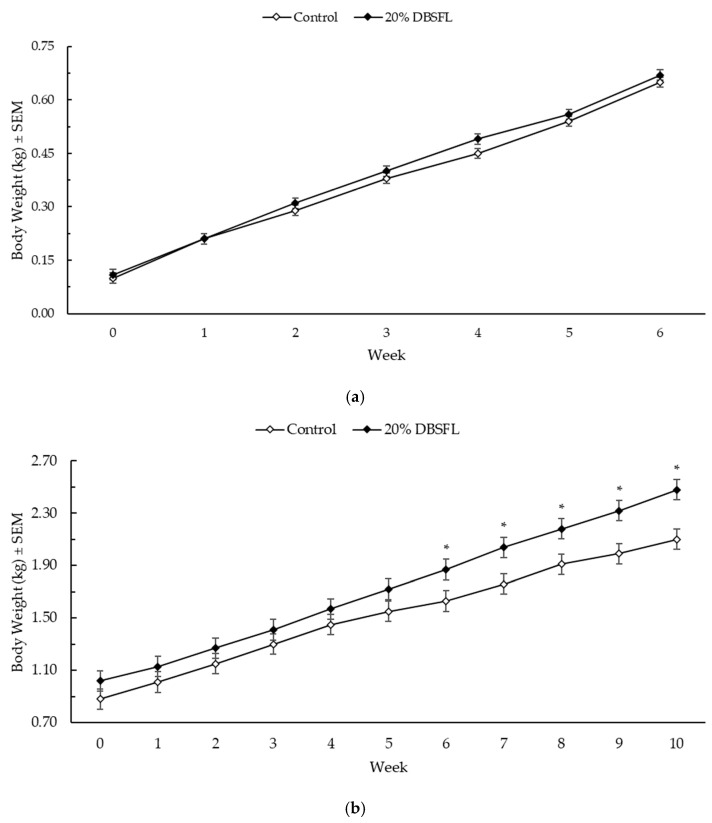
Kitten body weights during (**a**) nursing and (**b**) growth periods. Queens (*n* = 16) were fed control diets containing chicken meal and poultry fat or diets containing 20% dried whole black soldier fly larvae (DBSFL) through gestation and lactation periods. Kittens (all kittens born on trial for nursing period, *n* = 8 per dietary treatment for the post-weaning period, representing at least 3 unique litters) nursed from their dams until weaning and then were fed the same dietary treatment during the growth period. An * indicates a significant (*p* < 0.05) difference in body weight between diets.

All kittens were given a BCS of 5 (ideal) at the start, mid-point, and at end of the growth phase. The kittens’ body weights during lactation and the post-weaning period are shown in Figure 1. During the lactation phase, the kittens maintained similar body weights across treatments, with a trend for higher body weight in the kittens from the DBSFL-fed queens at week 4 of lactation (*p* = 0.06). During the growth phase, the kittens from the DBSFL-fed queens were significantly heavier at weeks 6 through 10 (*p* < 0.05 for each).

The sex of the kittens significantly impacted their body weight, where male kittens were heavier than females through to week 5 (*p* < 0.01), and kittens from larger litters were heavier than those from smaller litters (*p* < 0.05).

During the growth period, the kittens fed the DBSFL diet consumed significantly more than those receiving the control diet at week 5 (*p* = 0.04) and week 10 (*p* = 0.01) (Table 5). The energy intake, on a kcal per day basis, of the kittens fed the DBSFL diet was numerically greater than for those fed the control diets at week 5 and statistically greater at week 10 (*p* = 0.03). The fecal scores did not differ due to dietary treatment (Table 5).

The dry matter and crude protein digestibility levels were significantly higher in the kittens fed the DBSFL diet (*p* = 0.04, *p* < 0.0001, respectively, Table 6) relative to the controls. The ash and energy digestibility tended to be higher for the DBSFL diets than the control diet (*p* = 0.10, *p* = 0.07, respectively). The taurine digestibility was not affected by dietary treatment (*p* = 0.12).

In the absence of historical control data or reference ranges specific to kittens, the values for adult cats were used for comparative purposes, although it is unclear if these values for adult cats are appropriate for the evaluation of kitten hematology or blood chemistry, since differences have been seen due to the age of kittens versus adults [31].

During the kitten growth period, the blood hematology parameters were generally within the reference ranges described for adult cats, except for reticulocyte hemoglobin, which was lower than the adult reference range in both the control and DBSFL-fed groups (Table 7).

The dietary treatment was observed to impact several blood hematology parameters. Relative to the controls, several RBC numbers tended to be lower in the DBSFL-fed kittens at the beginning of the growth trial (*p* = 0.06) but were similar thereafter. Likewise, the RDW was significantly lower in the DBSFL-fed kittens at the beginning of the growth trial (*p* < 0.01) but was similar thereafter. The absolute number of reticulocytes tended to be higher in the DBSFL-fed kittens at the mid-point of the growth trial (*p* = 0.06) and was significantly higher at the end of the growth period (*p* = 0.04). The reticulocyte hemoglobin counts were also higher in the DBSFL-fed kittens at the beginning of the growth trial (*p* = 0.01) and at the end (*p* = 0.03). Furthermore, the WBC numbers were higher in the DBSFL-fed kittens at the mid-point of the growth trial compared to the control-fed kittens (*p* = 0.01). For blood clinical chemistry parameters with no significant impact or trend for an impact of diet, data are not presented.

Several serum chemistry parameters from the kittens on the growth trial were outside the adult cat reference ranges, including the SDMA, globulin, and ALP levels in both the DBSFL-fed kittens and controls (Table 8). For serum chemistry parameters with no significant impact or trend for an impact of diet, data are not presented. There were several parameters for which the dietary treatment had a significant impact. The blood glucose level was significantly lower in the DBSFL-fed kittens at baseline (*p* = 0.03) but was not different thereafter. The blood urea nitrogen level was significantly higher at the start and mid-point of the growth trial for the DBSFL-fed kittens compared to the control-fed kittens (*p* = 0.04, *p* = 0.01, respectively) and tended to be higher at the end of the growth period for the DBSFL-fed kittens (*p* = 0.08). The albumin level tended to be higher at the end of the growth period for the DBSFL-fed kittens (*p* = 0.07). The alkaline phosphatase the was significantly higher at baseline in the DBSFL-fed kittens compared to the control-fed kittens (*p* = 0.04). Finally, the triglyceride counts were significantly higher at baseline and at the end of the growth period in the DBSFL-fed kittens relative to the controls (*p* = 0.04, *p* = 0.05, respectively).

The whole blood taurine concentrations were not affected by diet or time (mean = 371.51 ± 13.177 nmol/mL), while the plasma taurine concentration was lower in the DBSFL-fed kittens at baseline (*p* = 0.03, Table 8) but was similar thereafter.

With respect to the urinalysis parameters in the kittens, there was no impact of dietary treatment on the urine pH or specific gravity (Table 9). The urinary creatinine level was significantly lower in the DBSFL-fed kittens compared to the control-fed kittens at the mid-point of the growth trial (*p* = 0.04) and tended to be lower at the end of the growth trial (*p* = 0.06). The urinary protein the tended to be lower in the kittens fed the DBSFL diet compared to those fed the control diet at the mid-point of the growth trial (*p* = 0.07).

## 4. Discussion

Together, the findings of the study demonstrate that DBSFL has the ability to sustain gestation, lactation, and growth; therefore, it is suitable for inclusion in the food of cats of all life stages. The design of the study followed the general principles laid down in the minimum feeding protocol for proving a gestation–lactation claim for a cat food (AAFCO, 2024 [27]). With this design, the queens must be in at least their second heat period and at least one year or age and the kittens are to be followed for at least the first 6 weeks of age, independent of when they are weaned. The AAFCO protocol was designed for complete food for cats but was considered relevant to this study on the basis that DBSFL and its components, defatted BSFL and BSFL oil, have the potential to be primary nutrient sources in cat food. Key differences were introduced to expand the value of the study, particularly the use of a control group rather than use of historical control colony data only, to allow a direct comparison of DBSFL with a diet containing conventional protein and fat sources, i.e., chicken meal and poultry fat. Additionally, the duration of the study of growth in kittens was extended from 6 to 10 weeks to include the most sensitive period of life and also that of the highest growth. These data further allow the bridging of maintenance data in adult dogs and cats (typically greater than 1 year old) in the published literature to these more demanding life stages.

Previous evaluations using the precision-fed cecectomized rooster assay suggested that DBSFL is a high-quality protein and amino acid source [13]. Similarly, in studies in adult cats and dogs, the digestibility of DBSFL meal has generally been similar to, or slightly below, poultry-based control diets [15,17,21]. This study provided an opportunity to evaluate the quality of the protein source in vivo under periods of high nutrient demand. The queens included in the trial had similar food intake, BW, fecal quality, and litter size levels when fed a DBSFL-based diet as compared to a chicken meal and poultry fat-based (control) diet, indicative of DBSFL being of sufficient quality to maintain health during gestation and lactation. The kittens nursing from the queens fed the DBSFL diet or directly consuming that diet post-weaning had higher dietary intake and BW levels and also had higher protein digestibility levels. Together, the improved growth performance and protein digestibility of the kittens fed DBSFL are indicative of the better palatability and nutrient availability of DBSFL when contributing significantly to the protein and energy levels of the diet compared to chicken meal and poultry fat. The basis for the improved nutrient digestibility seen in this trial may be related to the exposure to this ingredient at an early life stage, allowing for better adaptation of the developing gut microflora to BSFL-based nutrients. There is evidence of modulation of gut microflora with BSFL-based diets. For example, when 20% DBSFL meal was introduced into the diet of adult dogs, after one month there were significant changes observed in short-chain fatty acid concentration, microbial populations, and the interruption of a number of metabolic pathways relative to the controls fed a chicken-meal-based diet [17]. Additionally, on an as-fed basis, the control and DBSFL diets contained 0.01% and 2.08%, respectively, of lauric acid. The presence of lauric acid in this DBSFL ingredient may provide a readily available energy source in young animals for which gut development is not complete, and which has been demonstrated in other species [32]. Lauric acid has also been implicated in mammary gland development and milk fat production in ruminants [33], as well as improved gut morphology and epithelial cell turnover in weaned piglets [34,35]. Thus, there are several potential mechanisms by which DBSFL-based diets may contribute to improved nutrient availability.

There were a few differences in blood parameters between the DBSFL-fed and control groups that were most likely due to normal biological variation and the physiological adaptations to pregnancy, lactation, and rapid growth and not related to diet. In the queens, all values remained within the reference ranges, although the reticulocyte concentrations were variable during the trial in both dietary groups, with a significant difference between the controls and DBSFL-fed queens observed at mid-gestation. In cats, mature red blood cells serve as a primary source of iron for the developing fetus [36]. Thus, it is likely these changes are a result of the need to support the developing fetus and regenerate mature erythrocytes for the queen rather than any impact of dietary treatment. Additionally, the SDMA values were slightly above the reference ranges during the baseline and post-partum assessments in both dietary groups. Symmetric dimethylarginine is used as a marker of renal function, and elevated SDMA levels can be of concern [37]. However, in this trial, no other markers of renal function were elevated (e.g., creatinine, BUN, urinalysis), indicating normal physiological variation or a response to pregnancy rather than pathology. Moreover, the urinary protein levels were higher post-partum in the DBSFL-fed queens but the protein/creatinine ratios were also well within the normal ranges, consistent with normal renal function [38]. The additional variation in blood values among the groups of queens was also likely a consequence of pregnancy rather than an effect of diet.

In the kittens on the trial, several hematology and blood clinical chemistry values deviated from the standard reference ranges, although as these ranges were derived from adult cats and all kittens presented as healthy at the veterinary inspections, these differences are unlikely to be a cause for concern. For example, the ALP concentration was dramatically higher than the reference range but has been shown to be substantially higher in kittens and to decline as they reach adult body size [31]. Similarly, the creatinine concentration was lower than the reference range but this also is typical of growing kittens [31]. Higher numbers of reticulocytes and reticulocyte hemoglobin counts, as well as higher BUN concentrations, were generally observed throughout the growth period in kittens consuming the DBSFL diet relative to those fed the control diet. These findings are likely to reflect the nutritional differences of the two diets, although the changes were not considered to be biologically significant, falling well within the reference ranges. The data from this trial provide valuable additional information on the standard blood chemistry and hematology profiles of both kittens and queens during gestation and lactation.

The urinary creatinine and protein levels were lower in the kittens fed the DBSFL diet, although the kittens from both treatments had urinary protein/creatinine ratios in the normal range [38]. Protein sources can vary significantly in their creatinine content [39], and the availability to the animal is influenced by the protein digestibility and other factors [39,40,41]. Thus, these effects are attributed to a nutritional effect, with no biological relevance.

Finally, it is important to reiterate that the life stage, feedstock, and processing methods used to create BSFL-based ingredients can impact the nutrient profile and availability of those ingredients [12]. Amino acid profiles are largely taxon-based, and although the diet can influence the fatty acid profile, typically lauric acid is always present. Therefore, these nutrients from DBSFL are likely to be similar between sources provided the larvae are reared under controlled conditions using nutritionally balanced, feed-grade materials. However, other factors will contribute to the overall quality of the DBSFL, including the relative amounts of protein and fat, which are driven to some extent by the feedstock, the mineral profile, the presence of heavy metals, and other contaminants from the feedstock. Careful control of the production process is an established critical factor in the production of feed-grade DBSFL, and the source and rearing methods used to generate BSFL ingredients should be considered when extrapolating these results to other BSFL ingredients sources [12,42,43].

Considering the importance of alternative protein sources in the diet of cats, these data contribute not only to the knowledge of DBSFL as an ingredient but also provide valuable insight into the overall health of cats during gestation and lactation and of kittens pre- and post-weaning under controlled conditions in a study designed to meet AAFCO guidelines for proving the adequacy of complete food for these life stages. As mentioned above, the comparative data using the AAFCO feeding protocol are based on historical controls, and the results of the study above, particularly with regards to blood parameters, contribute to the body of knowledge under current feeding practices using conventional and novel protein sources.

## 5. Conclusions

Overall, these data are the first to demonstrate the safety and efficacy of DBSFL, as well as its protein and energy components, for the gestation and lactation of queens and for the growth of kittens. The nutritional profile, which in part reflects the feed of the larvae, potentially influences the value of DBSFL as a protein and energy source during these sensitive life stages. In this trial, the BSFL were fed a diet based on bakery materials and distillery by-products from the bourbon industry, resulting in a unique nutrient profile that may not be replicated with other husbandry and feeding programs. Recognizing that the rearing program used to create BSFL-derived ingredients may directly impact the suitability of the ingredient, care should be taken when evaluating other BSFL sources for these critical life stages of companion animals.

## Figures and Tables

**Table 1:** Nutrient profile of dried whole black soldier fly larvae. The DBSFL were derived from 4th–5th instar larvae raised on bakery materials and bourbon distillery by-products (EnviroFlight LLC, Maysville, KY, USA). All nutrients, except moisture, are presented on a dry matter basis.

Nutrient	Concentration
Proximate Composition (%)	
Moisture	3.46
Crude protein	42.00
Crude fat (acid hydrolysis)	36.20
Crude fiber	6.10
Acid detergent fiber	7.20
Neutral detergent fiber	15.10
Total dietary fiber	13.91
Insoluble dietary fiber	10.18
Soluble dietary fiber	3.69
Indispensable AAs (%)	
Arginine	1.73
Histidine	1.01
Isoleucine	1.54
Leucine	2.23
Lysine	2.18
Methionine	0.51
Phenylalanine	1.39
Threonine	1.25
Tryptophan	0.50
Valine	1.96
Selected dispensable AA (%)	
Cystine	0.28
Selected fatty acids (%)	
Lauric acid (C12:0, %)	12.74
Myristic acid (C14:0)	3.37
Palmitic acid (C16:0)	5.55
Oleic acid (C18:1)	5.76
Linoleic acid (C18:2)	6.40
Linolenic acid (C18:3)	0.55
Selected minerals	
Calcium (%)	1.49
Phosphorus (%)	0.81
Magnesium (%)	0.29
Sodium (%)	0.10
Potassium (%)	1.05
Iron (mg/kg)	135
Manganese (mg/kg)	110
Zinc (mg/kg)	85.7
Copper (mg/kg)	8.50

Abbreviations: AA: amino acid.

**Table 2:** Ingredient and nutrient compositions of treatment diets fed to queens.

	Diet Composition (%, As-Is)	
Ingredient	Control	20% DBSFL
Chicken meal	35.4	24.3
DBSFL	0.0	20.0
Corn	17.8	15.4
Brewers rice	17.8	15.4
Poultry fat	11.6	7.2
Dried egg whites	5.0	5.0
Corn gluten meal	3.6	3.6
Fish meal (Menhaden)	3.0	3.0
Dried beet pulp	3.0	3.0
Dry Digest Cat (palatant)	1.50	1.50
Potassium chloride	0.35	0.35
Salt	0.30	0.30
Choline chloride 60%	0.28	0.28
Calcium chloride	0.20	0.20
Vitamin premix ^1^	0.10	0.15
Dicalcium phosphate	0.10	0.10
Mineral premix ^2^	0.06	0.09
Taurine	0.05	0.06
Naturox Plus (Antioxidant)	0.03	0.03
Analyzed nutrient content (% dry matter basis)		
Dry matter	94.1	92.8
Crude protein	36.5	37.7
Crude Fat (acid hydrolysis)	19.6	18.0
Crude fiber	2.04	3.60
Ash	6.98	7.17
Phosphorus	1.08	0.97
Calcium	1.74	1.57
Arginine	2.35	2.66
Histidine	0.63	0.81
Isoleucine	1.25	1.55
Methionine	0.74	0.73
Cystine	0.46	0.41
Leucine	2.69	2.91
Lysine	2.16	2.37
Phenylalanine	1.31	1.61
Tyrosine	1.02	1.31
Threonine	1.01	1.12
Tryptophan	0.27	0.32
Valine	1.64	1.97
Lauric acid	0.01	2.24
Myristic acid	0.17	0.68
Palmitic acid	4.73	3.58
Stearic acid	1.19	0.84
Linoleic acid	3.84	3.59
Linolenic acid	0.20	0.37
Calculated ME (kcal/kg, [26])	4867	4735

Abbreviations: DBSFL: dried whole black soldier fly larvae; ME: metabolizable energy. ^1^ Vitamin premix supplied vitamin A (retinyl palmitate), vitamin D3 (cholecalciferol), vitamin E (alpha-tocopheryl acetate), thiamine (thiamin mononitrate 14,252 mg/kg), riboflavin (4719 mg/kg), pantothenic acid (12,186 mg/kg), Niacin (64,736 mg/kg), pyridoxine (5537 mg/kg), folic acid (720 mg/kg), biotin (70 mg/kg), and vitamin B12 (22 mg/kg). ^2^ Trace Mineral Premix supplied calcium (calcium carbonate, 20.5–24.6%), zinc (zinc sulfate, 8.8%), iron (ferrous sulfate, 3.8%), copper (copper sulfate, 1.1%), manganese (manganous oxide, 5684 ppm), selenium (sodium selenite, 310 ppm), and iodine (1584 ppm).

**Table 3:** Diet intake, body weight, and fecal characteristics of queens during gestation and lactation. Queens (*n* = 16) were fed control diets (based on chicken meal and poultry fat) or diets containing 20% dried whole black soldier fly larvae through the gestation and lactation periods.

		Diet			*p*-Value
Variable	Time ^1^	Control	20% DBSFL	SEM	Diet
Diet intake (g/day)	Week 1	33.78	29.97	7.460	0.719
	Mid-gestation	60.02	55.11		0.644
	Post-partum	73.95	71.72		0.833
	Weaning	137.27	116.50		0.058
Calculated energy intake (kcal ME/day)	Week 1	154.73	131.70	33.412	0.628
	Mid-gestation	274.87	242.23		0.492
	Post-partum	338.69	315.22		0.621
	Weaning	628.65	512.02		0.018
Body weight (kg)	Baseline	3.31	3.48	0.241	0.630
	Mid-gestation	3.65	3.75		0.767
	Post-partum	4.66	4.61		0.879
	Weaning	3.74	3.95		0.541
Fecal score	Baseline	2.31	2.50	0.123	0.288
	Mid-gestation	2.25	2.31		0.722
	Post-partum	2.31	2.65		0.069
	Weaning	2.51	2.58		0.738

Abbreviations: DBSFL: dried whole black soldier fly larvae; SEM: pooled standard error of the mean. ^1^ Baseline = week 0; mid-gestation = week 4; post-partum = week 9; weaning = week 15.

**Table 4:** Select blood hematology parameters of queens during gestation and lactation. Queens (*n* = 16) were fed control diets containing chicken meal and poultry fat or diets containing 20% dried whole black soldier fly larvae through gestation and lactation. Reference ranges are based on the laboratory reference ranges (IDEXX Laboratories Canada, Markham, ON, Canada).

		Diet			*p*-Value	Reference Range
Variable	Time ^1^	Control	20% DBSFL	SEM	Diet	
MCV (fL)	Baseline	40.0	41.8	0.69	0.092	39–56
	Mid-gestation	40.4	42.2		0.083	
	Post-partum	42.0	42.7		0.466	
	Weaning	39.7	41.8		0.048	
MCH (pg)	Baseline	14.0	14.7	0.27	0.075	12.6–16.5
	Mid-gestation	13.8	14.4		0.138	
	Post-partum	14.0	14.5		0.215	
	Weaning	13.5	14.2		0.070	
Reticulocytes (10^3^/μL)	Baseline	7.1	15.1	5.86	0.342	3–50
	Mid-gestation	13.0	30.2		0.046	
	Post-partum	13.2	21.4		0.330	
	Weaning	20.1	21.3		0.886	
Reticulocyte hemoglobin (pg)	Baseline	15.5	15.5	0.37	0.916	15.3–22.9
	Mid-gestation	14.8	15.3		0.327	
	Post-partum	14.9	15.8		0.090	
	Weaning	14.4	15.4		0.053	
WBC (10^9^/L)	Baseline	8.5	9.6	1.80	0.650	3.9–19.0
	Mid-gestation	11.4	11.5		0.957	
	Post-partum	13.6	13.7		0.981	
	Weaning	20.6	13.4		0.009	
Platelets (10^9^/L)	Baseline	341	288	31.1	0.239	155–641
	Mid-gestation	445	362		0.067	
	Post-partum	476	456		0.648	
	Weaning	439	344		0.041	

Abbreviations: DBSFL: dried whole black soldier fly larvae; MCH: mean corpuscular hemoglobin; MCV: mean corpuscular volume; SEM: pooled standard error of the mean; WBC: white blood cells. ^1^ Baseline = week 0; mid-gestation = week 4; post-partum = week 9; weaning = week 15.

**Table 5:** Kitten food intake post-weaning. Kittens (*n* = 8 per dietary treatment for the post-weaning period, representing at least 3 unique litters and sex-ratio-matched) nursed from their dams until weaning and then were fed the same dietary treatment as their dams (control diets with chicken meal and poultry fat or diets containing 20% dried whole black soldier fly larvae) during the growth period.

		Diet			*p*-Value
Variable	Time	Control	20% DBSFL	SEM	Diet
Diet intake (g/d)	Week 1	42.59	44.80	3.872	0.691
	Week 5	53.86	65.58		0.044
	Week 10	61.08	76.35		0.011
Calculated energy intake (kcal ME/d)	Week 1	195.09	196.89	17.351	0.942
	Week 5	246.70	288.21		0.105
	Week 10	279.75	335.55		0.033
Fecal score [29]	Week 1	2.69	2.50	0.147	0.373
	Week 5	2.60	2.69		0.676
	Week 10	2.88	2.75		0.551

Abbreviations: DBSFL: dried whole black soldier fly larvae; SEM: pooled standard error of the mean.

**Table 6:** Nutrient digestibility in kittens. Kittens (*n* = 8 per dietary treatment for the post-weaning period, representing at least 3 unique litters and sex-ratio-matched) nursed from their dams until weaning and then were fed the same dietary treatment (control diets with chicken meal and poultry fat or diets containing 20% dried whole black soldier fly larvae) during the growth period.

	Diet			*p*-Value
Nutrient	Control	20% DBSFL	SEM	Diet
Dry matter (%)	80.3	83.1	0.90	0.044
Crude protein (%)	75.4	84.5	1.02	<0.0001
Crude fat (%)	91.1	89.9	1.02	0.391
Crude fiber (%)	45.5	44.0	2.88	0.725
Ash (%)	37.8	44.4	2.60	0.100
Nitrogen-free extract (%)	91.2	90.5	0.69	0.248
Gross energy (%)	83.4	86.0	0.94	0.066

Abbreviations: DBSFL: dried whole black soldier fly larvae; SEM: pooled standard error of the mean.

**Table 7:** Blood hematology data from kittens during the growth period. Kittens (*n* = 8 per dietary treatment for the post-weaning period, representing at least 3 unique litters and sex-ratio-matched) nursed from their dams until weaning and then were fed the same dietary treatment (control diets with chicken meal and poultry fat or diets containing 20% dried whole black soldier fly larvae) during the growth period. Reference ranges are based on the laboratory reference ranges (IDEXX Laboratories Canada, Markham, ON, Canada).

		Diet			*p*-Value	Reference Range
Variable	Time	Control	20% DBSFL	SEM	Diet	
RBCs (10^12^/L)	Week 1	7.9	7.3	0.22	0.059	7.1–11.5
	Week 5	7.6	7.3		0.294	
	Week 10	8.5	8.1		0.216	
RDW	Week 1	25.6	20.9	0.73	0.0001	10–26
	Week 5	19.5	20.1		0.524	
	Week 10	19.7	19.5		0.838	
Reticulocytes (10^3^/μL)	Week 1	10.7	12.7	2.77	0.611	3–50
	Week 5	7.6	15.3		0.060	
	Week 10	10.8	19.3		0.039	
Reticulocyte hemoglobin (pg)	Week 1	14.1	15.2	0.26	0.011	15.3–22.9
	Week 5	14.5	14.5		0.944	
	Week 10	14.0	14.8		0.035	
WBCs (10^9^/L)	Week 1	13.2	11.8	0.97	0.296	3.9–19.0
	Week 5	10.0	13.5		0.014	
	Week 10	11.6	11.4		0.856	

Abbreviations: DBSFL: dried whole black soldier fly larvae; RBCs: red blood cells; RDW: red cell distribution width; SEM: pooled standard error of the mean; WBCs: white blood cells.

**Table 8:** Serum chemistry data from kittens during the growth trial. Kittens (*n* = 8 per dietary treatment for the post-weaning period, representing at least 3 unique litters and sex-ratio-matched) nursed from their dams until weaning and then were fed the same dietary treatment (control diets with chicken meal and poultry fat or diets containing 20% dried whole black soldier fly larvae) during the growth period. Reference ranges are based on the laboratory reference ranges (IDEXX Laboratories Canada, Markham, ON, Canada).

		Diet			*p*-Value	Reference Range
Variable	Time	Control	20% DBSFL	SEM	Diet	
Glucose (mmol/L)	Week 1	5.2	4.8	0.12	0.029	4.0–9.7
	Week 5	4.7	4.6		0.523	
	Week 10	4.4	4.5		0.722	
SDMA (µg/dL)	Week 1	19.6	20.1	1.20	0.770	0–14
	Week 5	17.3	17.5		0.884	
	Week 10	18.5	16.4		0.219	
Urea (BUN) (mmol/L)	Week 1	7.1	8.4	0.41	0.039	5.7–13.2
	Week 5	8.3	9.8		0.013	
	Week 10	8.2	9.3		0.075	
Albumin (g/L)	Week 1	30.3	30.5	0.66	0.792	26–39
	Week 5	30.4	29.6		0.430	
	Week 10	29.9	31.6		0.071	
Globulin (g/L)	Week 1	20.5	19.6	1.05	0.559	30–59
	Week 5	24.8	24.0		0.616	
	Week 10	27.3	25.3		0.185	
ALP (IU/L)	Week 1	126	156	9.8	0.040	12–59
	Week 5	145	168		0.107	
	Week 10	132	152		0.144	
Triglycerides (mmol/L)	Week 1	0.25	0.34	0.030	0.035	0.23–1.03
	Week 5	0.34	0.32		0.700	
	Week 10	0.29	0.38		0.046	
Plasma taurine (nmol/mL)	Week 1	116.4	105.1	15.01	0.025	80–120
	Week 5	119.8	132.6		0.548	
	Week 10	119.4	144.5		0.245	

Abbreviations: ALP: alkaline phosphatase; BUN: blood urea nitrogen; DBSFL: dried whole black soldier fly larvae; SDMA: symmetric dimethylarginine; SEM: pooled standard error of the mean.

**Table 9:** The urinalysis from kittens during the growth phase. Kittens (*n* = 8 per dietary treatment for the post-weaning period, representing at least 3 unique litters and sex-ratio-matched) nursed from their dams until weaning and then were fed the same dietary treatment (control diets with chicken meal and poultry fat or diets containing 20% dried whole black soldier fly larvae) during the growth period.

		Diet			*p*-Value
Variable	Time	Control	20% DBSFL	SEM	Diet
Urine pH	Week 1	5.97	5.93	0.194	0.903
	Week 5	6.19	6.22		0.896
	Week 10	6.15	6.22		0.794
Specific gravity	Week 1	1.05	1.05	0.004	0.846
	Week 5	1.06	1.06		0.366
	Week 10	1.06	1.06		0.574
Creatinine (µmol/L)	Week 1	15,983	12,585	1520.4	0.122
	Week 5	19,511	14,819		0.035
	Week 10	21,503	17,387		0.063
Urinary protein (mg/dL)	Week 1	39.88	39.25	0.194	0.900
	Week 5	48.88	39.75		0.074
	Week 10	41.63	35.38		0.216

## Data Availability

The original contributions presented in this study are included in the article or Supplementary Material. Further inquiries can be directed to the corresponding author.

## Supplementary Materials

The following supporting information can be downloaded at https://www.mdpi.com/article/10.3390/ani15081078/s1: Table S1. Reference ranges for parameters evaluated in queens through gestation and lactation (IDEXX Laboratories Canada, Markham, ON, Canada). Table S2. Blood clinical chemistry of queens during gestation and lactation. Queens (*n* = 16) were fed control diets containing chicken meal and poultry fat or diets containing 20% dried whole black soldier fly larvae through gestation and lactation. Table S3. Urinalysis of queens during gestation and lactation. Queens (*n* = 16) were fed control diets containing chicken meal and poultry fat or diets containing 20% dried whole black soldier fly larvae through gestation and lactation.

## Abbreviations

The following abbreviations are used in this manuscript:

AA	Amino acid
AAFCO	Association of American Feed Control Officials
ALP	Alkaline phosphatase
ALT	Alanine transaminase
ANOVA	Analysis of variance
AST	Aspartate aminotransferase
BCS	Body condition score
BSFL	Black soldier fly larvae
BUN	Blood urea nitrogen
BW	Body weight
DBSFL	Dried whole black soldier fly larvae
DM	Dry matter
GGT	Gamma-glutamyl transferase
MCH	Mean corpuscular hemoglobin
MCHC	Mean corpuscular hemoglobin concentration
MCV	Mean corpuscular volume
ME	Metabolizable energy
Na/K	Sodium/potassium
NFE	Nitrogen-free extract
NRC	National Research Council
RBC	Red blood cell
RDW	Red cell distribution width
SDMA	Symmetric dimethylarginine test
UPCR	Urine protein creatinine ratio
WBC	White blood cell
