# Supplementary material for: Dried Whole Black Soldier Fly Larvae Consumption Supports Gestation, Lactation, and Growth in Cats
Source: Animals (Basel). 2025 Apr 8;15(8):1078. doi: 10.3390/ani15081078 (PMC12023938; doi:10.3390/ani15081078)
Supplement: Supplementary file 1 [file animals-15-01078-s001.zip › animals-3517594-supplementary.pdf]

Table S1. Reference ranges for parameters evaluated in queens through gestation and lactation (IDEXX Laboratories Canada, Markham, Ontario, Canada).

Parameter	Reference Range	Parameter	Reference Range
RBC ($x10^{12}$/L)	7.1 - 11.5	Glucose (mmol/L)	4.0 - 9.7
Hematocrit (L/L)	0.29 - 0.45	SDMA (μg/dL)	0 - 14
Hemoglobin (g/L)	103 - 162	Creatinine (μmol/L)	80 - 203
MCV (fL)	39 - 56	Urea (BUN) (mmol/L)	5.7 - 13.2
MCH (fL)	12.6 - 16.5	Phosphorus (mmol/L)	0.9 - 2.0
MCHC (g/L)	285 - 378	Calcium (mmol/L)	2.2 - 2.7
RDW	10 - 26	Magnesium (mmol/L)	0.70 - 1.00
Reticulocyte ($x10^3$/μL)	3 - 50	Sodium (mmol/L)	147 - 157
Reticulocyte Hgb (pg)	15.3 - 22.9	Potassium (mmol/L)	3.7 - 5.2
WBC ($x10^9$/L)	3.9 - 19.0	Na:K ratio	29 - 42
Neutrophils ($x10^9$/L)	2.6 - 15.2	Chloride (mmol/L)	114 - 126
Lymphocytes ($x10^9$/L)	0.9 - 5.9	Bicarbonate (mmol/L)	12 - 22
Monocytes ($x10^9$/L)	0.0 - 0.5	Total protein (g/L) [1]	63 - 88
Eosinophils ($x10^9$/L)	0.0 - 2.2	Albumin (g/L)	26 - 39
Basophils ($x10^9$/L)	0.0 - 0.1	Globulin (g/L)	30 - 59
Platelets ($x10^9$/L)	155 - 641	Albumin:globulin ratio	0.5 - 1.2
		ALT (IU/L)	27 - 158
		AST (IU/L)	16 - 67
		ALP (IU/L)	12 - 59
		GGT (IU/L)	0 - 6
		Bilirubin – total	0.0 - 5.2
		Bilirubin - conjugated	0.0 - 3.4
		Cholesterol (mmol/L) [2]	2.4 - 7.9
		Triglycerides (mmol/L)	0.23 - 1.03
		Amylase (IU/L)	623 - 2,239
		Lipase C	0 - 45
		Creatine kinase (IU/L)	64 - 440

Abbreviations: ALP: alkaline phosphatase; ALT: alanine transaminase; AST: aspartate aminotransferase; BUN: blood urea nitrogen; GGT: gamma-glutamyl transferase; MCH: mean corpuscular hemoglobin; MCHC: mean corpuscular hemoglobin concentration; MCV: mean corpuscular volume; Na:K: sodium:potassium; RBC: red blood cells; RDW: red cell distribution width; SDMA: symmetric dimethylarginine; WBC: white blood cells. [1] Total protein was outside of normal ranges for adult cats fed the control-diet at post-partum (61.2 g/L). [2] Cholesterol was outside of normal ranges for adult cats fed the control diet at weaning (1.90 mmol/L) and the DBSFL-diet at post-partum (2.39 mmol/L) and weaning (2.16 mmol/L).

Table S2. Blood clinical chemistry of queens during gestation and lactation. Queens (n=16) were fed control diets containing chicken meal and poultry fat, or diets containing 20% dried whole black soldier fly larvae (DBSFL) through gestation and lactation.

Variable	Time[1]	Diet		SEM	*p*-value	Reference Range
		Control	20% DBSFL		Diet	
Glucose, mmol/L	Baseline	4.3	4.3	0.24	0.942	4.0 - 9.7
	Mid-gestation	4.3	4.3		0.892	
	Post-partum	3.5	3.7		0.585	
	Weaning	4.2	4.1		0.843	
SDMA, μg/dL	Baseline	15.1	14.9	0.88	0.843	0 - 14
	Mid-gestation	11.5	13.1		0.233	
	Post partum	17.0	17.8		0.552	
	Weaning	11.9	11.9		0.972	
Globulin, g/L	Baseline	33.8	36.3	1.43	0.225	30 - 59
	Mid-gestation	34.5	38.2		0.093	
	Post partum	29.1	33.6		0.033	
	Weaning	32.3	34.8		0.253	
ALP, IU/L	Baseline	23.3	25.1	3.71	0.723	12 - 59
	Mid-gestation	19.8	18.6		0.835	
	Post-partum	25.3	26.6		0.795	
	Weaning	36.4	46.9		0.059	

Abbreviations: ALP: alkaline phosphatase; DBSFL: dried whole black soldier fly larvae; SDMA: symmetric dimethylarginine; SEM: pooled standard error of the mean. [1] Baseline = week 0; mid-gestation = week 4; post-partum = week 9; weaning = week 15.

Table S3. Urinalysis of queens during gestation and lactation. Queens (n=16) were fed control diets containing chicken meal and poultry fat, or diets containing 20% dried whole black soldier fly larvae (DBSFL) through gestation and lactation.

Variable	Time[1]	Diet		SEM	*p*-value	Reference Range[2]
		Control	20% DBSFL		Diet	
Urine pH	Baseline	6.65	6.46	0.139	0.344	6 - 7.5
	Mid-gestation	6.69	6.37		0.100	
	Post-partum	6.61	6.38		0.251	
	Weaning	6.60	6.37		0.260	
Specific gravity	Baseline	1.06	1.06	0.004	0.500	1.001 - 1.085
	Mid-gestation	1.08	1.07		0.341	
	Post partum	1.07	1.06		0.947	
	Weaning	1.07	1.07		0.649	
Creatinine (μmol/L)	Baseline	37,126	36,761	3,564.9	0.943	
	Mid-gestation	34,234	33,240		0.845	
	Post partum	32,154	31,781		0.943	
	Weaning	35,292	31,353		0.452	
Urinary protein (mg/dL)	Baseline	39.88	43.38	8.087	0.761	
	Mid-gestation	50.50	48.88		0.888	
	Post-partum	40.45	65.00		0.043	
	Weaning	36.59	36.50		0.994	

Abbreviations: DBSFL: dried whole black soldier fly larvae; SEM: pooled standard error of the mean. [1] Baseline = week 0; mid-gestation = week 4; post-partum = week 9; weaning = week 15. [2] Yadav, S.N.; Ahmed, N.; Nath, A.J.; Mahanta, D.; Kalita, M.K. Urinalysis in dog and cat: A review. *Vet World* **2020**, *13*, 2133-2141, doi:10.14202/vetworld.2020.2133-2141.
